# Cell Adhesion Molecules Affected by Ionizing Radiation and Estrogen in an Experimental Breast Cancer Model

**DOI:** 10.3390/ijms232012674

**Published:** 2022-10-21

**Authors:** Gloria M. Calaf, Leodan A. Crispin, Juan P. Muñoz, Francisco Aguayo, Gopeshwar Narayan, Debasish Roy

**Affiliations:** 1 Instituto de Alta Investigación, Universidad de Tarapacá, Arica 1000000, Chile; 2 Laboratorio de Oncovirología, Programa de Virología, Instituto de Ciencias Biomédicas (ICBM), Facultad de Medicina, Universidad de Chile, Santiago 8380000, Chile; 3 Department of Molecular and Human Genetics, Banaras Hindu University, Varanasi 221005, India; 4 Department of Natural Sciences, Hostos College of the City University of New York, Bronx, NY 10451, USA

**Keywords:** ionizing radiation, cell adhesion, breast cancer, E-cadherin, desmocollin, gap junction protein, Integrins, Keratins, Laminins

## Abstract

Cancer develops in a multi-step process where environmental carcinogenic exposure is a primary etiological component, and where cell–cell communication governs the biological activities of tissues. Identifying the molecular genes that regulate this process is essential to targeting metastatic breast cancer. Ionizing radiation can modify and damage DNA, RNA, and cell membrane components such as lipids and proteins by direct ionization. Comparing differential gene expression can help to determine the effect of radiation and estrogens on cell adhesion. An in vitro experimental breast cancer model was developed by exposure of the immortalized human breast epithelial cell line MCF-10F to low doses of high linear energy transfer α particle radiation and subsequent growth in the presence of 17β-estradiol. The MCF-10F cell line was analyzed in different stages of transformation that showed gradual phenotypic changes including altered morphology, increase in cell proliferation relative to the control, anchorage-independent growth, and invasive capability before becoming tumorigenic in nude mice. This model was used to determine genes associated with cell adhesion and communication such as E-cadherin, the desmocollin 3, the gap junction protein alpha 1, the Integrin alpha 6, the Integrin beta 6, the Keratin 14, Keratin 16, Keratin 17, Keratin 6B, and the laminin beta 3. Results indicated that most genes had greater expression in the tumorigenic cell line Tumor2 derived from the athymic animal than the Alpha3, a non-tumorigenic cell line exposed only to radiation, indicating that altered expression levels of adhesion molecules depended on estrogen. There is a significant need for experimental model systems that facilitate the study of cell plasticity to assess the importance of estrogens in modulating the biology of cancer cells.

## 1. Introduction

Cancer develops in a multi-step process where environmental carcinogen exposure is a primary etiological component, and where cell adhesion governs the biological activities of tissues [1]. Despite advances in breast cancer treatment, patients with this cancer often relapse and develop metastasis, which accounts for over 90% of the 458,000 breast cancer-related deaths [2] and it has become the most commonly diagnosed (11.7%) disease worldwide in 2020 [3]. Tumor metastasis is a complex and intricate process by which tumor cells disseminate from primary tumors to secondary ones [4]. Identifying the molecular genes that regulate this process is essential to targeting metastatic breast cancer.

The breast epithelium depends on interactions between epithelial cells and the extracellular matrix with the underlying stroma that works as a unit that continually communicates with each other for their structural integrity and specific function [5,6].

There are two forms of radiation such as non-ionizing and ionizing. Non-ionizing radiation can come from a variety of natural sources, such as the sun and lighting, as well as man-made sources, such as those utilized in industrial/medical applications and wireless communications [7]. Ionizing radiation is made up of electrically charged particles (ions), both positive and negative, such as alpha particles and electrons [8]. Alpha and beta radiation are two of the most well-known ionizing radiations; alpha particles are formed by two neutrons and two protons from the nucleus of the atom during the decrease of the atomic mass number and reduction of the atomic number; it results from the radioactive decay of heavy elements such as plutonium, radium, or thorium, and their weight prevents them from traveling far, and although these particles cannot travel through paper or human skin; if they are introduced into the body from a radioactive source, they can damage cells and DNA [9]. When beta particles are released during radioactive decay, they are negatively charged, unlike alpha particles [10]. Even though these particles can travel greater distances, they can be stopped by a thin coating of substance; yet, if ingested or inhaled, the damage can be as severe as that produced by alpha particles [10,11]. Other types of ionizing radiation include gamma radiation [10], X-ray radiation [9,10], and cosmic radiation, which enters the atmosphere and mostly consists of protons, alpha particles, and heavier atomic nuclei [12].

Ionizing radiation has the unique property of randomly penetrating different tissues and cells, harming them according to the dose absorbed rather than just by the cells exposed [13]. By direct ionization or water radiolysis, ionizing radiation can change and damage DNA, RNA, and cell membrane components like lipids and proteins [14]. Water radiolysis involves several reactive oxygen species (ROS), a mechanism that has been identified as the primary cause of cell death and tissue damage [15,16]. However, it was postulated that a certain degree of alterations was proposed to the self-correction capacity of the DNA that might lead to mutations that could potentially be part of carcinogenic processes [17]. The research on radiation-induced effects and their biological repercussions has become more complicated due to the emergence of dynamic signaling pathways [14].

Another factor associated with radiation is hypoxia, a hallmark of solid tumors; it means that is a major obstacle to the effectiveness of radiation therapy; thus, authors [18] have shown that hypoxia induces autophagy and its effects on the response of breast cancer cells to ionizing radiation correspond to a marked accumulation of autophagosomes accompanied by mRNA induction of the autophagy-related genes. Such autophagy has been associated with increased radioresistance of tumor cells. A blockade of autophagy contributed to the retardation of DNA double-strand break, repair, and significant radiosensitization indicating that suppression of autophagy is useful for therapies related to hypoxic breast cancer cells by ionizing radiation [19,20,21].

Transcriptional regulators have been identified to induce epithelial-to-mesenchymal transition (EMT) which plays an important role in development and malignancy [22]. EMT-inducing transcription factors and dysregulated proteins related to them have been investigated such as E-cadherin, which has a pivotal role in epithelial cell behavior, tissue formation, and suppression of cancer, and Cadherin cell–cell adhesion proteins are critical for the formation of tissues from single cells [23].

Cadherins are hormonally controlled and play a physiological role throughout mammary development; however, when these genes are altered, they might lead to pathological effects [22]. Then, the role of desmosomal cadherins and their downstream signaling events are important in the malignant behavior of breast cancers [24]. On the other hand, desmosomes play a crucial role in cell–cell adhesion since they are sites of adhesion between adjacent cells in layers of epithelial and some non-epithelial tissues, playing an important role in the maintenance of tissue structure, and losing these components leads to a lack of adhesion and a gain of cellular mobility [25].

The desmocollin 3 gene *DSC3* is a member of the cadherin superfamily of calcium-dependent cell adhesion molecules, an important component of desmosomes and its expression is down-regulated in breast cancer cell lines and primary breast tumors [26].

The gap junction protein, alpha 1 gene *GJA1* overexpression has been linked to a poor prognosis in several human cancers; hence the role of gap junction intercellular communication (GJIC) during anchorage-independent that occurs from the earliest stages of cancer cell aggregation in MCF7 breast cancer cell line is important [27].

Keratins (cytokeratins) are intermediate filament proteins expressed in a differentiation status-specific manner in luminal (K7, K8, K18, K19) or basal (K5, K6, K14, K17) epithelial cells and are routinely used as diagnostic markers for cancer tissues [28].

Invasive lobular and ductal carcinomas have been reported to lose or down-regulate ligand *laminin 5* (*LN5*), a heterotrimeric laminin protein exclusive to epithelial cells that comprise three polypeptide chains produced by three distinct encoding genes such as the laminin alpha 3 gene *LAMA3*, the laminin beta 3 gene *LAMB3*, and *LAMC2* [29]. The normal epithelial uses hemidesmosomes to adhere to the basement membrane and the major structural proteins of the hemidesmosomes are the Integrin and its ligand *LN5* [30].

The extracellular proteins, Laminins, and their transmembrane receptors, the Integrins, belong to the Kyoto Encyclopedia of Genes and Genomes (KEGG) pathway concerning cell communication, cell adhesion, and cell surface receptor link and are involved in two-way signaling, crucial for generating signals that regulate both form and function of specific tissues [31].

This work aimed to analyze genes involved in cellular adhesion induced by the effect of ionizing radiation and hormones such as estrogen in non-tumorigenic and tumorigenic cell lines derived from the experimental breast cancer model including (i) differential gene expression; (ii) gene expression levels correlating tumor and normal tissues across various cancer types; (iii) comparison between gene expression and estrogen receptor status in TCGA breast cancer; and (iv) clinical relevance of gene expression related to cell adhesion across various cancer types analyzed by the disease stage factor.

## 2. Results

### 2.1. Experimental Design of a Radiation and Estrogen-Induced Breast Cancer Model

This work is based on a previously developed experimental human breast cancer model where a normal human breast epithelial cell line transformed into a malignant one by the effect of ionizing radiation and estrogens [1]. Some genes associated with cell adhesion were analyzed from this radiation- and estrogen-induced experimental breast cancer model, including E-cadherin gene *CDH1*, *DSC3*, *GJA1*, the Integrin alpha 6 gene *ITGA6*, the Integrin beta 6 gene *ITGB6*, the Keratin genes *KRT14*, *KRT16*, *KRT17*, *KRT6B*, and *LAMB3*.

The cell lines used in this model were: (i) the parental cell line MCF-10F (C); (ii) an Estrogen cell line (E), MCF-l0F continuously grown with estradiol; (iii) a malignant and non-tumorigenic cell line named Alpha3 (A3); (iv) a malignant and tumorigenic cell line named Alpha5 (A5), and (v) the Tumor2 cell line (T2) derived from a xenograft of the A5 cell line injected into nude mice [1].

### 2.2. Analysis of Differential Gene Expression in an Induced-Radiation and -Estrogen Experimental Breast Cancer Model

Gene expression microarrays have been an effective tool for comparing and contrasting cell lines and disease states in people [32,33]. Various microarray studies have identified similarities and differences in mRNA expression levels among samples; thus biological annotations such as Gene Ontology (GO) or KEGG pathways have been utilized in studies to extrapolate biological roles [34,35] and regulatory relationships from changes in individual genes [36,37]. To further investigate the biological functions of identified differentially expressed genes; the GO and KEGG functional enrichment analysis tools revealed that up-regulated genes were enhanced, whereas others were down-regulated as those involved in cell cycle regulation [38].

**Figure 1:**
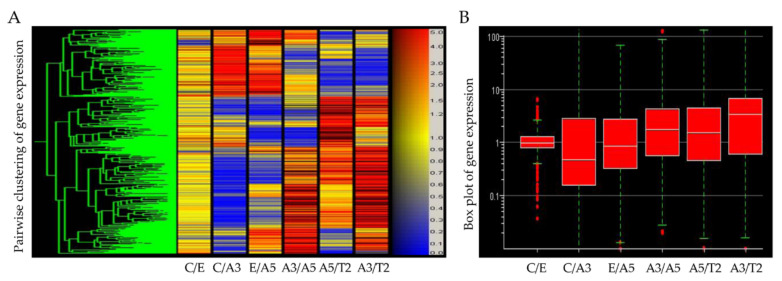
(**A**) Heatmap of Affymetrix array (U133A) data that allows comparing the gene expression of the cell lines derived from the model: MCF-10F/Estrogen (C/E); control/Alpha3 (C/A3); estrogen/Alpha5 (E/A5); Alpha3/Alpha5 (A3/A5); Alpha5/Tumor2 (A5/T2), and Alpha3/Tumor2 (A3/T2) (Reprinted/adapted with permission from Ref. [39]. 2013, Spandidos Publications). The red color indicates a higher expression; blue, a lower expression, and yellow equal expression (**left panel**). (**B**) The gene box plot summarizes the range of differential gene expression in the same pairwise cell line comparisons (**right panel**).

Differentially expressed genes obtained from an Affymetrix array U133A indicated that high LET radiation such as that emitted by radon progeny, in the presence of estrogen, induced a cascade of events indicative of cell transformation and tumorigenicity in human breast epithelial cells (Figure 1). The studies reported in this work are associated with genes involved in the control of changes in cellular adhesion components that take place by the effect of radiation and estrogen in the normal non-tumorigenic MCF-10F and the tumorigenic cell lines. As it was seen in this model the T2 cell line was developed from the MCF-10F, a normal cell line, non-tumorigenic in the immune-suppressed nude or SCID mice.

### 2.3. Gene Expression in a Breast Cancer Model

Profiling of differentially expressed genes related to cell adhesion was studied with genes obtained through an Affymetrix array (U133A) such as (A) the E-cadherin gene *CDH1*, (B) the desmocollin 3 gene *DSC3*, (C) the gap junction protein, alpha 1 gene *GJA1*, (D) (a) the Integrin alpha 6 gene *ITGA6*, (b) the Integrin beta 6 gene *ITGB6*, (E) the laminin beta 3 gene *LAMB3*, and (F) the Keratin genes (a) *KRT14*, (b) *KRT16*, (c) *KRT17*, and (d) *KRT6B* in the following cell lines: MCF-10F/Estrogen (C/E); Control/Alpha3 (C/A3); Estrogen/Alpha5 (E/A5); Alpha3/Alpha5 (A3/A5); Alpha5/Tumor2 (A5/T2) and Alpha3/Tumor2 (A3/T2).

**Figure 2:**
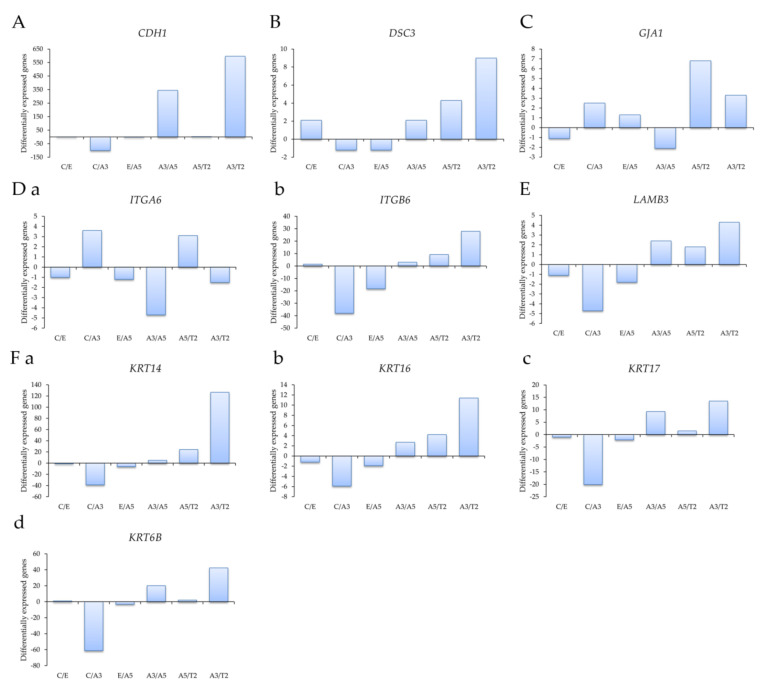
Profiling of differentially expressed genes obtained through an Affymetrix array (U133A) data comparing (**A**) the E-cadherin gene *CDH1*, (**B**) the desmocollin 3 gene *DSC3*, (**C**) the gap junction protein, alpha 1 gene *GJA1*, (**D**) (**a**) the Integrin alpha 6 gene *ITGA6*, (**b**) the Integrin beta 6 gene *ITGB6*, (**E**) the laminin beta 3 gene *LAMB3*, and (**F**) the Keratin genes (**a**) *KRT14*, (**b**) *KRT16*, (**c**) *KRT17*, and (**d**) *KRT6B* in the following cell lines: MCF-10F/Estrogen (C/E); Control/Alpha3 (C/A3); Estrogen/Alpha5 (E/A5); Alpha3/Alpha5 (A3/A5); Alpha5/Tumor2 (A5/T2) and Alpha3/Tumor2 (A3/T2). The graphs were obtained from a Cluster-dendrogram repository of gene expression from our laboratory for this article.

Affymetrix array (U133A) data indicated that *CDH1* gene expression levels were higher in the tumor T2 cell line than in the A3 cell line, non-tumorigenic (Figure 2A). Thus, cell adhesion molecules were expressed at higher levels in malignantly transformed breast epithelial cells derived from athymic animals relative to levels of non-tumorigenic cells as the A3 cell line that was treated only by ionizing radiation. The tumorigenic A5 cell line was also higher than the A3 cell line. There was a non-significant difference between A5 and T2, neither E and A5 nor C and E.

Results in Figure 2B show that *DSC3* gene expression levels were higher in T2 than in A3 cell lines and A5 cell lines; C and A5 cell lines had higher DSC3 gene expression than A3 cell lines. However, there was no significant difference among other groups. *GJA1* gene expression levels were higher in the T2 cell line than in the A3 and A5 cell lines in Figure 2C. There was a non-significant difference between the other groups. Figure 2D shows that (a) *ITGA6* gene expression levels were higher in the A3 cell line than in the A5, T2, and C cell lines; its levels were higher in the T2 cell line than in the A5 cell line. It also shows that the T2 cell line had higher (b) *ITGB6* expression levels than the A3 and A5 cell lines, and also higher in the C than the A3 cell line. There was a non-significant difference between the other groups. Figure 2E shows that LAMB3 expression levels were higher in the T2 cell line than in the A3 cell line and C than A3 cell lines. There was a non-significant correlation between the other groups.

Results in Figure 2F showed that (a) *KRT14*, (b) *KRT16*, (c) *KRT17*, and (d) *KRT6B* gene expression levels were higher in the T2 than the A3 cell line and higher in the C cell line than the A3 cell line.

### 2.4. Differential Gene Expression Levels between Tumor and Normal Tissues across Various Cancer Types

**Figure 3:**
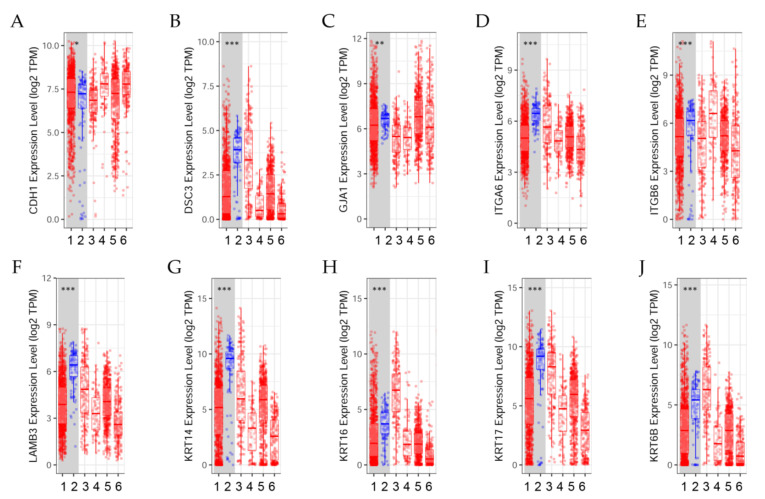
Differential gene expression levels between tumor and normal tissues across various cancer types. The box plots show the distribution of gene expression levels of (**A**) *CDH1*, (**B**) *DSC3*, (**C**) *GJA1*, (**D**) *ITGA6*, (**E**) ITGB6, (**F**) *LAMB3*, (**G**) *KRT14*, (**H**) *KRT16*, (**I**) *KRT17*, and (**J**) *KRT6B* in tumors versus normal tissues (Wilcoxon test, *: *p* < 0.05, **: *p* < 0.01; ***: *p* < 0.001) estimated by TIMER2.0 in breast invasive carcinoma [41]. (1) BRCA.Tumor (*n* = 1093), (2) BRCA.Normal (*n* = 112), (3) BRCA-Basal.Tumor (*n* = 190), (4) BRCA-Her2.Tumor (*n* = 82), (5) BRCA-LumA.Tumor (*n* = 564), (6) BRCA-LumB.Tumor (*n* = 217).

The differential expression between tumor and adjacent normal tissues of genes across TCGA breast tumors is presented in Figure 3. Distributions of gene expression levels are displayed using box plots. The statistical significance computed by the Wilcoxon test is annotated by the number of stars (*: *p*-value < 0.05; **: *p*-value < 0.01; ***: *p*-value < 0.001). Comparing differential gene expression can help determine the effect of radiation and estrogens on cell adhesion, a process that governs the biological behaviors of cells [40].

Results indicated that (A) *CDH1* expression level was significantly (*p* < 0.05) higher in tumors than in normal breast tissues; (B) *DSC3* expression was significantly (*p* < 0.001) higher in normal tissues than in tumors. (C) *GJA1* expression level was significantly (*p* < 0.01) higher in normal tissues than in tumors. (D) *ITGA6* and (E) *ITGB6* expression levels were significantly (*p* < 0.001) higher in normal tissues than in tumors.

The differential gene expression levels between tumor and adjacent tissue displayed in the box plot (Figure 3F–J) showed that *LAMB3*, *KRT14*, *KRT16*, *KRT17*, and *KRT6B* expression levels were significantly (*p* < 0.001) higher in normal tissues than in tumors.

### 2.5. Gene Expression and Estrogen Receptor Status in TCGA Breast Cancer

The Cancer Genome Atlas (TCGA), a collaboration between the National Cancer Institute (NIH) and National Human Genome Research Institute (NHGRI), has generated comprehensive, multi-dimensional maps of the key genomic changes in 33 types of cancer and completes the most comprehensive cross-cancer analysis to date [42]. UCSC Xena is an online exploration tool for public and private, multi-omic and clinical/phenotype data [43].

**Figure 4:**
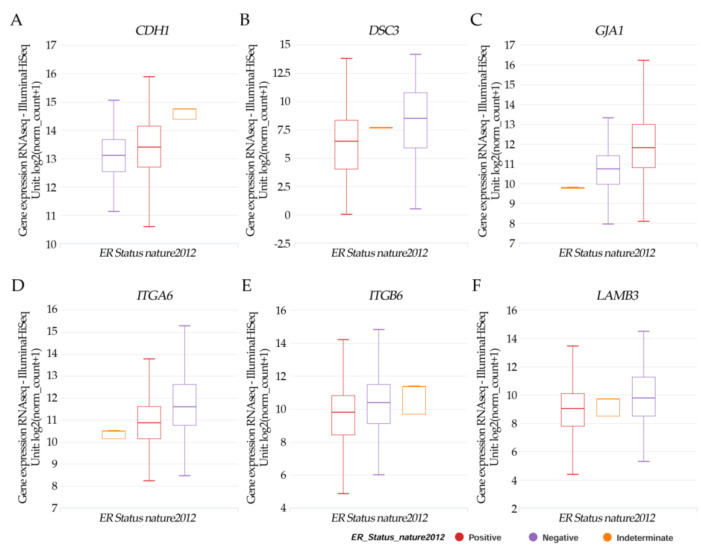
Xena Chart View showing box plot transcript expressions of (**A**) *CDH1*, (**B**) *DSC3*, (**C**) *GJA1*, (**D**) *ITGA6*, (**E**) *ITGB6*, and (**F**) *LAMB3* in breast cancer (cohort: TCGA Breast Cancer (BRCA), *n* = 1247) stratified by nature2012 for estrogen receptor status (One-way ANOVA, *p* < 0.05). Raw data were extracted from the University of California, Santa Cruz (http://xena.ucsc.edu/ (accesses on 20 August 2021)). UCSC Xena functional genomics explorer (https://xenabrowser.net accessed on 20 August 2021) [43].

Results in Figure 4A–F from the UCSC Xena online exploration tool indicated that the patients’ samples having (A) *CDH1* had higher positive ER expression but there was no significance. (B) *DSC3*; (D) *ITGA6*, (E) *ITGB6*; and (F) *LAMB3* had a significant (*p* < 0.05) higher negative ER expression. However, (C) *JGA1* had a significant (*p* < 0.05) higher positive ER expression.

**Figure 5:**
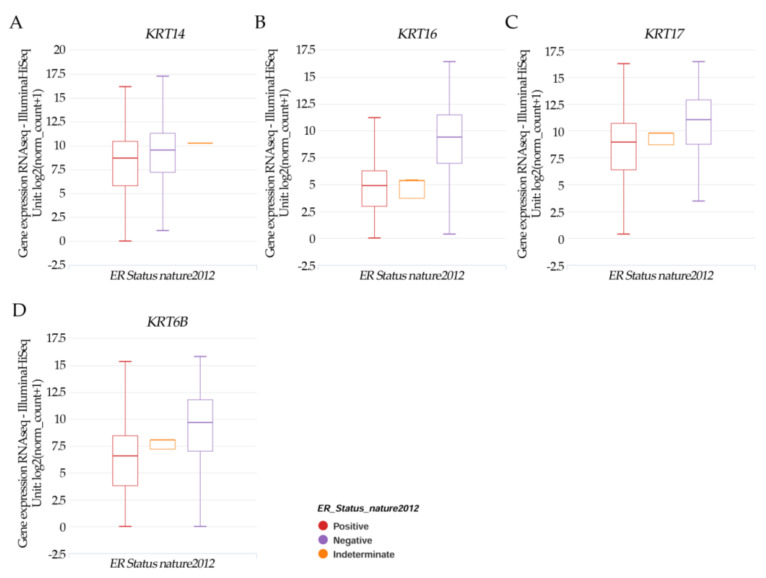
Xena Chart View showing a box plot transcript expression of the keratin genes (**A**) *KRT14*, (**B**) *KRT16*, (**C**) *KRT17*, and (**D**) *KRT6B* in breast cancer (cohort: TCGA Breast Cancer (BRCA), *n* = 1247) stratified by nature2012 for estrogen receptor status (One-way ANOVA, *p* < 0.05). Raw data were extracted from the University of California, Santa Cruz (http://xena.ucsc.edu/ (accesses on 20 August 2021)). UCSC Xena functional genomics explorer (https://xenabrowser.net accessed on 20 August 2022) [43].

Results in Figure 5A–D indicated that the patients’ samples having *KRT14*, *KRT16*, *KRT17*, and *KRT6B* had a significant (*p* < 0.05) higher negative ER expression.

### 2.6. Clinical Relevance of Gene Expression across Various Cancer Types Analyzed by the Disease Stage Factor

The molecular constituents of cell–cell communication pathways have been identified thanks to extensive genetic analysis research [44]; and the identification of genes associated with a specific tissue has been useful for highlighting their biological function, providing context for disease states, like breast cancer and subtype as BRCA-Basal, BRCA-Her2, BRCA-Lum-A, and BRCA-Lum-B. Cell adhesion molecules such as alpha, beta, and gamma catenins, cadherins, desmocolin/desmoglein, Integrin alpha6, Integrin beta2, and LAMB3 among others are important in these processes.

Overall, when the clinical stages of patients were analyzed (Table 1 and Table 2) in breast invasive carcinoma (BRCA), results indicated that the *CDH1 DSC3*, *GJA1*, *ITGA6*, *ITGB6*, *LAMB3*, *KRT14*, *KRT16*, *KRT17*, and *KRT6B* gene expression levels were significantly (*p* < 0.001) higher in stages 3 and 4 in all BRCA patients than in other clinical stages (Table 1). Their expression levels were non-significant in any of the stages in BRCA-Basal patients; There was a significant (*p* < 0.01) or (*p* < 0.05) difference in stage 4 in BRCA–Her2 patients; a significant (*p* < 0.001) or (*p* < 0.01) difference in stage 4 in BRCA-LumA patients. Their expression levels were significant (*p* < 0.01) in stage 4 in BRCA-LumB patients except for *KRT14* (*p* < 0.05), and additionally, *CDH1*, *DSC3*, and *JGA1* expressions were also significant (*p* < 0.05) in stage 3 in BRCA-LumB patients.

*CDH1* gene expression was significantly (*p* < 0.001) higher in stages 3 and 4 in all BRCA patients than in other clinical stages and Luminal A breast cancer subtype; but a non-significant difference in BRCA-Basal patients; a significant (*p* < 0.01) or (*p* < 0.05) difference in stage 4 in BRCA–Her2 and in stage 3 and 4 in BRCA-LumB patients.

*DSC3* gene expression level was significantly (*p* < 0.001) higher in both stages 3 and 4 in all BRCA and stage 4 in BRCA-LumA patients than in any other clinical stages (Table 1). There was also a significant (*p* < 0.01) difference in stage 4 in BRCA-LumB patients, and a significant (*p* < 0.05) difference in stage 4 in BRCA-Her2 and stage 3 in BRCA-LumB patients; however, this expression level was not significant in BRCA-Basal patients.

*GJA1* gene expression level was significantly (*p* < 0.001) higher in both stages 3 and 4 in all BRCA and stage 4 in BRCA-LumA patients than in any other clinical stages (Table 1). There was a significant (*p* < 0.01) difference in stage 4 in BRCA-Her2 and BRCA-LumB patients, and a significant (*p* < 0.05) difference in stage 3 in BRCA-LumB patients; however, this expression level was not significant in BRCA-Basal patients.

*ITGA6* and *ITGB6* gene expression levels were significantly (*p* < 0.001) higher in both stages 3 and 4 in all BRCA and stage 4 in BRCA-LumA patients than in any other clinical stages (Table 1). They also had a significant (*p* < 0.01) difference in stage 4 in BRCA-LumB patients, and a significant (*p* < 0.05) difference in stage 4 in BRCA-Her2 patients; however, their expression levels were not significant in BRCA-Basal patients.

The clinical relevance of *LAMB3* gene expression level was significantly (*p* < 0.001) higher in both stages 3 and 4 in all BRCA and stage 4 in BRCA-LumA patients than in any other clinical stages (Table 1). There was a significant (*p* < 0.01) difference in stage 4 in BRCA-Her2 and BRCA-LumB patients; however, this expression level was not significant in BRCA-Basal patients.

Results also indicated that *KRT14*, *KRT16*, *KRT17*, and *KRT6B* had significantly (*p* < 0.001) higher differences in stages 3 and 4 than in other clinical stages in all BRCA and *KRT16* in BRCA-LumA patients; a non-significant difference in BRCA-Basal in all patients; a significant (*p* < 0.01) or (*p* < 0.05) difference in stage 4 in BRCA–Her2, BRCA-LumA, except for *KRT16*, and BRCA-LumB patients.

## 3. Discussion

This experimental breast cancer model based on the effect of ionizing radiation and estrogens allows us to analyze adhesion molecules that are involved in important biological processes in epithelial cells that change from normal to cancer cells contributing to disease progression [45]. Multiple biological activities are frequently linked to single genes or groups of related genes, and they usually correspond to differences in gene expression across different tissue types. [46].

The *CDH1* gene is linked to E-cadherin, a transmembrane glycoprotein that is a hallmark of epithelial-to-mesenchymal transition. Cell–cell adhesion is mediated by members of the cadherin-catenin system and among them, E-cadherin and β-catenin are important adhesion molecules for epithelial cell function and preservation of tissue integrity. According to previous work [39] related to cDNA expression, the analysis revealed elevated levels of gene expression involved in the cell adhesion function as α-catenin, β-catenin, γ-catenin, besides E-cadherin and Integrin in A5 and T2 cell lines relative to the non-tumorigenic MCF-10F, E, and A3 cell lines. The E and A3 cell lines did not show β-catenin protein expression and they were not tumorigenic. However, the A5 cell line was developed in the presence of radiation and estrogen combined and showed higher β-catenin protein expression and tumorigenic characteristics than the previous one. Under these conditions, the A5 cell line gave rise to mammary gland tumors and the T2 cell line is one of them.

This study corroborated analysis done by Calaf et al. (2013) where fold-change and pair-wise analysis of differentially expressed genes in a breast cancer model indicated that E-cadherin gene expression levels were highly expressed in T2 in comparison with A5 and A3 [39], this is probably due to the estrogen effect in the initiation process. The findings of Calaf’s (2013) study revealed that in the presence of estrogen, environmental factors such as ionizing radiation can have a significant impact on human breast cell adhesion phenomena, boosting or supporting the molecular events of cellular transformation.

The present study indicated that *CDH1* gene expression was higher in the T2 cell line than in the A3 cell line indicating that cell adhesion molecules were expressed at higher levels in malignantly transformed breast epithelial cells derived from the athymic animal after the A5 cell line injection than in a non-malignant cell such as the A3 cell line, induced only by double doses of ionizing radiation. Such results seem to indicate that the effect of estrogen that originated in the tumor altered expression levels of adhesion molecules, or tumor tissue microenvironment as suggested by others [39].

There was a non-significant correlation between *CDH1* gene expression and ER status, neither between BRCA-Basal and *CDH1* expression, a cell type very aggressive and capable of inducing metastasis. However, *CDH1* was greater in the BRCA–Her2, BRCA-LumA, and BRCA-LumB and in stages 3 and 4 than in other clinical stages in all BRCA patients. Furthermore, breast cancer subtype analysis showed a significant difference in *CDH1* expression between tumors and adjacent normal tissues.

E-cadherin is consistently expressed in various epithelial cancers and down-regulation or loss of E-cadherin expression in cancers arising from E-cadherin positive tissues as well as those arising from E-cadherin negative tissues is linked to cancer progression and may reflect tumor dedifferentiation [47,48,49]; thus, studies have indicated that the inactivation of this gene results in larger tumors, higher tumor grades, and an increased risk of metastasis and chemoresistance [47,48,49]. Thus, authors [50] have demonstrated multiple mechanisms that disrupt E-cadherin function in cancer such as inactivation of somatic and germline mutations, and epigenetic silencing by DNA methylation, among others. In breast cancers, the expression, or lack thereof, of E-cadherin can differentiate tumor types [50].

Authors [51] indicated that deregulation of E-cadherin complexes had a role in cancer progression since most solid tumors were epithelial in origin, indicating that cancer progression might be promoted by E-cadherin loss, mutation, or destabilization due to loss of p120 binding, hence evidence suggested that the E-cadherin/catenin complex was required for epithelial monolayer homeostasis and maintenance [51].

The primary epithelial cadherin, E-cadherin, is found at AJs, which are areas of cell–cell interaction and E-cadherin plays an essential role in the maintenance of epithelial integrity [52]. Regarding the inconsistencies between the lack of expression of E-cadherin in severe invasive carcinomas and desmosome activity, the relationship between E-cadherin presence/absence and the integrity of the desmosomes at the same level is crucial [53].

Overall, it is clear that a variety of mechanisms caused the loss or disruption of mature AJs at the apical ZA, and that the loss of anti-tumorigenic E-cadherin signaling, combined with the gain of nuclear catenin signaling and the activation of various additional pathways (Rho GTPases, PI3K), were key events in tumor progression and metastasis [51].

A study [54] reported reduced E-cadherin expression associated with high grade, triple-negative receptor status, reduced overall survival in invasive breast carcinoma, and triple-negative receptor status in lobular breast cancer. In such a study, it was demonstrated that estrogens played a key role in the formation and advancement of human malignancies, notably breast cancer progression, which was dependent on the malignant instability of AJs and tissue integrity disruption.

Malignant cells from breast tumors can exhibit a variety of phenotypic alterations in comparison with normal cells, hence, junctional complexes, which include adhesion belts and desmosomes, play a role in such phenotypic alterations in membrane and sub-membrane protein expression [53]. Cadherins are cell adhesion molecules that participate in these two types of junctions and they are classified as either classic cadherins (E-cadherin in epithelial adhesion belts) or non-classic cadherins (cadherins in the desmosomes) such as desmogleins (1 to 4) and desmocollins (1 to 3) that form the transmembrane contact area [53,55]; thus, desmosomal proteins maintain tissue architecture and losing these components leads to a lack of adhesion and a gain of cellular mobility. They may also interfere in tumor progression; hence authors have studied their regulation by estrogens in normal mammary cells and human breast cancer [56].

*DSC3* is a component of desmosomes, and its expression is down-regulated in breast cancer cell lines and primary breast tumors [26]. On the other hand, studies have indicated a negative significant difference between ER statuses and *DSC3* levels since expression levels were higher in normal tissues than in tumors, indicating a possible tumor suppressor gene function. Authors demonstrated that desmosomal cadherins, such as *DSC* and *DSG* are a group of adhesion molecules with a role in invasion and motility [57]. Among the genes related to desmosomes are Desmocollin (*DSC*) 1–3 and Desmoglein (*Dsg*), which are transmembrane proteins of the cadherin family that form the adhesive core of desmosomes [58]. The present result showed that *DSC3* gene expression levels were higher in the T2 cell line than A3 and A5 cell lines, indicating that altered expression levels of adhesion molecules depend on the tissue microenvironment of the tumor formed in the animal. According to other studies, the DSC1 is validated as a protein connected with the lymph node status of breast cancer luminal A patients and positive for Her-2 status, as demonstrated by immunohistochemistry in primary breast tumors [59].

*GJA1* gene expression levels were higher in the T2 cell line than A3 and A5 cell lines indicating the possible effect of estrogen and other factors from the tumor environment. On the other hand, *GJA1* expression was higher in tumors than in adjacent normal tissues; and a positive ER status when compared to *JGA1* gene expression in breast cancer patients from the cohort.

Another study showed a downregulation of the desmosomal cadherin desmocollin 3 in human breast carcinoma cell lines using cDNA microarray analysis [56]. In comparison to normal breast tissues, authors reported higher GJB2 gene expression in invasive ductal carcinoma of the breast and very intense GJB2 gene expression in most estrogen receptor (ER) negative breast cancer tissues; on the contrary, most ER-positive breast cancer samples exhibited weak staining that was not statistically significant in comparison to normal tissue.

Integrins are involved in regulating cellular adhesion and invasion and they play a pivotal role in cell migration [60]. Among the Integrins, the *ITGA6* and *ITGB6* are involved in biological processes related to cell-matrix adhesion and cell communication. Results reported here showed that *ITGA6* gene expression levels were higher in the A3 non-tumorigenic cell line than in the A5 cell line indicating the effect of radiation on the cellular adhesion process. However, *ITGB6* expression levels were higher in the T2 cell line than in the A3 cell line indicating that adhesion molecules depended on the effect of the characteristic of tumors, formed by A5 that was cultured in the presence of estrogen. It is known that invasive carcinomas begin with invasion and destruction of the basement membrane, although in situ carcinomas are intraepithelial; tumor cells become motile as they progress from an in situ to an invasive phenotype, breaking up their tight connections with adjacent cells and making their way through obstacles such as basement membrane and dense interstitial mesenchyme [61,62]. A previous study showed changes in the structure and composition of the basement membrane during the malignant transformation of the breast epithelium and progression to malignancy [6]. On the other hand, when analyzing differential gene expression of *ITGA6* and *ITGB6*, higher gene expression levels were observed in tumors than in normal adjacent tissues, considering them good markers for such patients. However, the ER status was negative in such patients when compared with *ITGA6* and *ITGB6* gene expression. The ITGA6 and ITGB6 gene expression levels were higher in both stages 3 and 4 in all BRCA and stage 4 in BRCA-LumA patients; there was a significant difference in stage 4 in BRCA-LumB patients as well as in BRCA-Her2 patients than in other clinical stages.

Results showed that LAMB3 expression levels were higher in the T2 cell line than in the A3 cell line indicating the effect of the combination of ionizing radiation and estrogen on such expression. Furthermore, analysis of *LAMB3* expression in adjacent normal tissues than in tumors of the breast across various cancer types of patients indicated that such correlation between *LAMB3* gene expression and ER status. Concerning the clinical relevance of LAMB3 gene expression levels, it was reported higher expression in both stages 3 and 4 in all BRCA and stage 4 in BRCA-Her2, BRCA-LumA, and -LumB patients than in any other clinical stages. However, this expression level was non-significant in BRCA-Basal patients.

The role of *LAMB3* in several biological processes such as cell adhesion, cell surface-receptor, and cell communication is important since those changes are highly uncontrolled in tumor cells; the loss of epithelial characteristics is typically observed late in human cancer progression and is correlated with acquiring invasive and metastatic potential [30].

Altered keratin expression is linked to changes in cancer cell shape and motility [63]. Results showed that *KRT14* and *KRT16* gene expression levels were higher in the T2 cell line than in the A3 cell line indicating that ionizing radiation and estrogen affected such expression levels of adhesion molecules. However, KRT17 and KRT6B gene expressions were higher in A3 than C cell lines indicating the effect of ionizing radiation alone. A significant negative correlation between ER status and *KRT14*, *KRT16*, *KRT17*, and *KRT6B* was observed indicating that the presence of keratins could be a good marker for breast carcinogenesis instead of ER status alone.

The differential expression between tumor and adjacent tissues expression levels displayed for *KRT14*, *KRT16*, *KRT17*, and *KRT6B* indicated that their expression levels were higher in normal tissues than in tumors. The *KRT14*, *KRT16*, *KRT17*, and *KRT6B* gene expression levels were higher in both stages 3 and 4 in all BRCA, and in stage 4 in BRCA-Her2, BRCA-LumA, and BRCA-LumB patients; and there was no significant difference in BRCA-Basal patients than in other clinical stages. Thus, a small subgroup of breast carcinomas expressed basal keratins, while the majority express luminal keratins. It is important to mention that those tumors with higher tumor grade, worse prognosis, shorter relapse-free, and overall survival are all linked to basal keratin expression [64,65,66]. When compared to luminal subtypes, basal-like breast carcinomas express both basal and luminal keratins and are associated with aggressive clinical behavior and a higher rate of metastasis, then invasion occurs in K14-positive cells required for metastases [67,68].

Immunohistochemistry has helped to answer the dilemma of clinic-pathological characteristics of breast cancer having limited accuracy in predicting survival. KR17 expression [69] for example, has been linked to triple-negative status such as ER/progesterone receptor/human epidermal growth factor receptor-2 [HER2] and decreased survival; hence, when KR17 protein and mRNA expression were compared to ER/progesterone receptor/HER2 receptor status and event-free survival, authors observed significant correlations not only among the mRNA levels of KRT17, but also KRT16, and KRT14 that predicts poor survival. High levels of KRT14 have been found in basal-like breast cancer as well as in the basal layer of the stratified squamous epithelium [70].

The analyses presented here exhibited a potential correlation of adhesion molecules with clinical outcomes. Furthermore, it also indicates the gene expression related to cell adhesion in cancer progression. These characteristics could be a significant target to interfere with the development of cancer. For this reason, cell adhesion molecules are suggested to be an effective biomarker as well as a potential therapeutic target in the effort to prevent breast cancers in humans.

## 4. Materials and Methods

*Cell lines*: MCF-10F cells were grown in DMEM/F-12 (1:1) medium supplemented with antibiotics [100 U/mL penicillin, 100 μg/mL streptomycin, 2.5 μg/mL amphotericin B (all from Life Technologies, Grand Island, NY, USA)] and 10 μg/mL and 5% equine serum (Biofluids, Rockville, MD, USA), 0.5 μg/mL hydrocortisone (Sigma, St. Louis, MO, USA) and 0.02 μg/mL epidermal growth factor (Collaborative Research, Bedford, MA, USA) were added [71,72,73,74].

*Alpha-model*: Exponentially growing MCF-10F cells were plated 3 days before irradiation at a density of 3 × 10^5^ cells in 60 mm diameter stainless steel rings with a 6 μm mylar bottom. Cells were irradiated with graded doses of 150 KeV/μm ^4^He ions accelerated with the 4 Me V van de Graaff accelerator at the Columbia University Radiological Research Facilities, as described previously [1]. These high-energy particles have a LET value comparable to the α particles emitted by radon progeny. MCF-10F cells irradiated with either a single or double dose of 30, 60, or 100 cGy of ^4^He ions were prepared by subculturing for 10–15 passages and 12–14 weeks between doses. Irradiated cultures were subcultured immediately to determine growth kinetics and expanded in culture to assay for transformed phenotypes and, at the same time, samples were frozen as future stock. The remaining cells were then sampled for various transformed phenotypes and further passaged for additional radiation treatment. Cells were subsequently cultured in the presence or absence of E. Irradiated cultures were assayed for cell growth kinetics, anchorage-independent growth, invasiveness, tumorigenicity, and determination of BRCA1, BRCA2, and RAD51 protein expression.

MCF-10F was analyzed in different stages of transformation after being irradiated with either a single 60 cGy dose or 60/60 cGy doses of alpha particles [1]. The cell lines used in this model were: (a) the parental cell line MCF-10F (Control); (b) an estrogen cell line (E), MCF-l0F continuously grown with estradiol (Sigma-Aldrich) at 10^−8^; (c) a malignant and non-tumorigenic cell line (60/60 cGy) named Alpha3 (A3); (d) a tumorigenic cell line (60/60 cGy plus estrogen) named Alpha5 (A5); and (e) the Tumor2 cell line (T2) derived from a xenograft of the Alpha5 cell line injected into nude mice. These cell lines were cultured in the presence or absence of estrogen for periods of up to 10 months (Copyright permission from reference [39]), the scheme of animals injected with the cell lines indicated that E and Alpha3 did not form mammary tumors; however, A5 and Tumor 2 were tumorigenic in the model and SCID animal.

*Affymetrix HG-U133A Plus 2.0 GeneChip microarray gene expression analysis*: The breast cancer model (Alpha-model) consists of: (i) MCF-10F, (ii) Estrogen, (iii) Alpha3, (iv) Alpha5, and (v) Tumor2 cell lines used to analyze gene expression by the Affymetrix U133A oligonucleotide microarray (Affymetrix, Santa Clara, CA, USA). Arrays were quantitatively analyzed for gene expression using the Affymetrix GeneChip Operating Software (GCOS) with a dual global scaling option in the Genes@Work software platform of the discovery algorithm SPLASH (structural pattern localization analysis by sequential histograms) with a false discovery rate of 0.05 [75]. The Affymetrix U133A oligonucleotide microarray experiment was done once and contained14.500 genes.

*Gene expression analysis and statistical analysis*. TIMER2.0, a web source of information, systematically evaluates the clinical impact of different immune cells in various cancer types through three components: Immune Association, Cancer Exploration, and Immune Estimation, each with several modules such as the Gene module in the Immune Association component that provided the statistical analysis carried out by Spearman’s p test. The Cancer Exploration component has the Gene_DE module that provided statistical analysis carried out by the Wilcoxon test; The Gene_Outcome module with the analysis carried out by the Z-Score test, and the Gene_Corr module provided the statistical analysis carried out by the Spearman’s test [41]. The clinical relevance of gene expression across various cancer types (Table 1 and Table 2) was provided by the Timer2.0 Gene_Outcome module [41]. This module uses Cox proportional hazard model to evaluate the outcome significance of gene expression, adjusted by the stage clinical factors.

UCSC Xena, another web source of information, provided the statistical significance computed by the One-way Anova test. UCSC Xena web source allows users to explore functional genomic data sets for correlations between genomic and/or phenotypic variables and focuses on integrative visualization of multi-omics datasets across different genomic contexts, including genes, genomic elements, or any genomic region, for both coding and non-coding parts of the genome [43]. A *p* < 0.05 was considered significant.

## 5. Conclusions

Overall, this study supports the idea that the expression of adhesion molecules could be used as the prognostic biomarker for the early detection of human breast cancer cases. In this study, molecular signatures that play key roles in cell adhesion were targeted. To determine the role of adhesion molecules as potential prognostic markers there is a significant need for experimental model systems. Such a model would facilitate the study of this plasticity and the importance of the adhesion state in modulating the biology of cancer cells. Such an experimental breast cancer model allowed us to analyze genes associated with cell adhesion that could help to improve the clinical outcome of chemotherapy. However, in vitro studies are not sufficient to explain in vivo situations. For that reason, in cancer, prognostic factors are important for early diagnosis as well as to get an efficient treatment to prevent the risk of overtreatment. Cell dependency must be considered in future treatment planning and the molecular and clinical features are important for radiotherapy. Thus, using gene technology and molecular information is possible to improve therapies and reduction of side effects. Therefore, these findings will provide new insight into breast cancer-related fields.

## Figures and Tables

**Table 1:** Clinical relevance of gene expressions across various cancer types analyzed by the disease stage factor.

Breast Cancer	*CDH1*	*DSC3*	*GJA1*	*ITGA6*	*ITGB6*	*LAMB3*
BRCA (*n* = 1100)	3, 4 ***	3, 4 ***	3, 4 ***	3, 4 ***	3, 4 ***	3, 4 ***
BRCA-Basal (*n* = 191)	N.S.	N.S.	N.S.	N.S.	N.S.	N.S.
BRCA-Her2 (*n* = 82)	4 **	4 *	4 **	4 *	4 *	4 **
BRCA-LumA (*n* = 568)	4 ***	4 ***	4 ***	4 ***	4 ***	4 ***
BRCA-LumB (*n* = 219)	3 *, 4 **	3 *, 4 **	3 *, 4 **	4 **	4 **	4 **

The statistical significance is annotated by the number of stars (*: *p*-value < 0.05; **: *p*-value < 0.01; ***: *p*-value < 0.001); 3, 4: clinical stage factor; N.S.: not significant. Data estimated by TIMER2.0 in breast invasive carcinoma [41].

**Table 2:** Clinical relevance of *KRT14*, *KRT16*, *KRT17,* and *KRT6B* gene expression levels across various cancer types analyzed by the disease stage factor.

Breast Cancer	*KRT14*	*KRT16*	*KRT17*	*KRT6B*
BRCA (*n* = 1100)	3, 4 ***	3, 4 ***	3, 4 ***	3, 4 ***
BRCA-Basal (*n* = 191)	N.S.	N.S.	N.S.	N.S.
BRCA-Her2 (*n* = 82)	4 **	4 *	4 **	4 *
BRCA-LumA (*n* = 568)	4 **	4 ***	4 **	4 **
BRCA-LumB (*n* = 219)	4 *	4 **	4 **	4 **

The statistical significance is annotated by the number of stars (*: *p*-value < 0.05; **: *p*-value < 0.01; ***: *p*-value < 0.001); 3, 4: clinical stage factor; N.S.: not significant. Data estimated by TIMER2.0 in breast invasive carcinoma [41].

## Data Availability

TIMER2.0 is freely available at http://timer.cistrome.org (accessed on 6 August 2021); UCSC Xena online exploration tools are freely available at http://xena.ucsc.edu/ (accesses on 20 August 2021).
